# Recurrent Ameloblastic Fibroma: Report of a Rare Case

**DOI:** 10.1155/2013/565721

**Published:** 2013-05-21

**Authors:** Ravikumar S. Kulkarni, Amitabh Sarkar, Sandeep Goyal

**Affiliations:** Department of Oral Pathology, Surendera Dental College and Research Institute, H. H. Gardens, Sri Ganganagar, Rajasthan 335001, India

## Abstract

Ameloblastic fibroma (AF) is an uncommon mixed neoplasm of odontogenic origin frequently seen in the second decade of life. It mainly presents as an intrabony lesion but can even occur peripherally. Histologically, our case showed hypercellular areas, an uncommon feature seen in typical AF. Whether this benign lesion is treated by mode of enucleation and curettage or by extensive surgery is still a topic of debate. An extensive surgical treatment is suggested as the initial approach due to its high recurrence rate (18%) and the greater chances of recurrent AFs transforming into ameloblastic fibrosarcoma (45%), together with a long-term followup. We report a case of recurrent AF with hypercellular ectomesenchyme which developed a year after its conservative removal. We conclude that in recurrent AF sufficient sections of the pathological specimen are to be taken to rule out any malignant changes that might have begun in focal areas.

## 1. Introduction 

Ameloblastic fibroma (AF) is a rare tumor of odontogenic origin comprising 1.5–4.5% of all odontogenic tumors [1]. It was first described by Kruse (1891) and later classified as a separate entity by Thoma and Goldman (1946) [1, 2].

Although reported in a wide age range (0.5–62 years), most AFs are seen in the first two decades of life with 77.7% of cases being diagnosed before the age of 20. Males show slightly higher prevalence (M : F = 1.4 : 1), and the posterior mandible is the most common anatomic location [2].

Small lesions are asymptomatic, whereas larger ones may cause painless swelling [2]. Approximately, 20% of cases are discovered accidentally on radiographs taken to detect the failure of tooth eruption [3–5]. Three-fourth cases are associated with impacted or unerupted teeth or at times develop in areas of congenitally missing teeth [2].

Radiologically, AF is a unilocular (when small) or multilocular (when large) radiolucent lesion often with a smooth, sclerotic border and may or may not produce bulging of bone [2, 6].

Grossly, it appears as a solid, soft tissue mass with a smooth surface. It may or may not be encapsulated [7]. Histologically, the ectomesenchymal portion is made up of primitive connective tissue, characterized by plump fibroblasts and delicate collagen fibrils closely resembling the dental papilla. The epithelial component which resembles embryonic dental lamina is arranged in various patterns—thin long strands, cords, nests, or islands. The strands show double or triple layer of cuboidal cells, in contrast to the nests which are surrounded by columnar ameloblast-like cells enclosing stellate reticulum-like cells. Cyst formation within the epithelium is uncommon [2, 6].

AF needs to be differentiated from ameloblastoma, odontogenic myxoma, dentigerous cyst, odontogenic keratocyst, central giant cell granuloma, and histiocytosis [8]. Presence of numerous mitotic cells or any atypical mitosis should suggest malignant entities such as AFS in the differential diagnosis [3, 4].

Treatment of AF in general is a conservative approach, such as enucleation with curettage of the surrounding bone along with the removal of the affected tooth [1, 3]. Long-term followup is necessary [4].

## 2. Case Report

**Figure 1:**
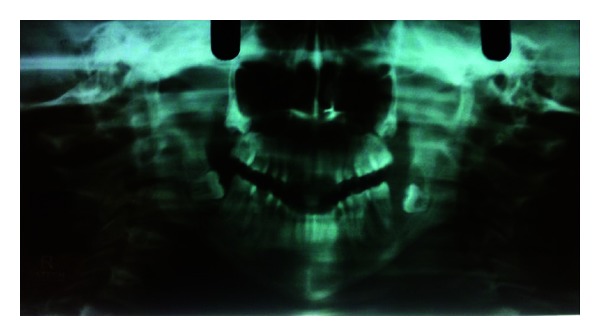
OPG showing well-defined radiolucency associated with an impacted mandibular left third molar (August 2011).

In August 2011, an 18-year-old female patient reported with a complaint of gingival overgrowth in the left lower back region, which interfered with chewing. Growth was insidious, slowly progressing, and no history of associated pain. On intraoral examination, a huge exophytic growth measuring roughly 3 × 4 cms was noticed posterior to the mandibular left second molar. It extended anteroposteriorly from 37 to retromolar pad, buccally in the vestibule and inferiorly till the floor of the mouth. On radiographic examination, a well-defined radiolucency was found around impacted 38 (Figure 1). Surgical excision of the entire intraosseous and extraosseous mass along with the removal of the impacted 38 was performed. A histopathologic diagnosis of ameloblastic fibroma was made. The patient was kept on followup. 

**Figure 2:**
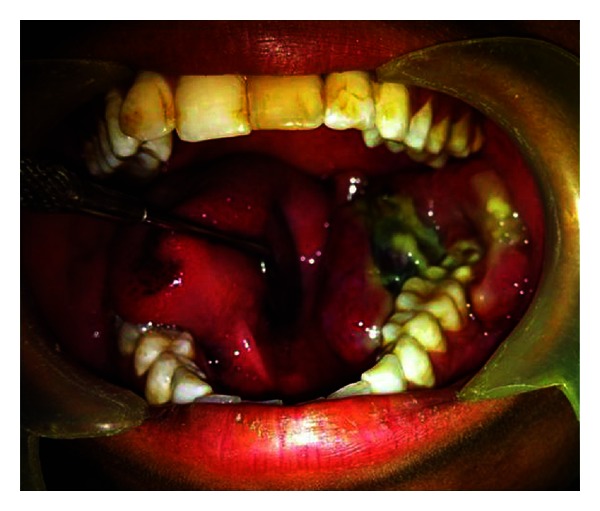
Intraoral view of tumor mass (August 2012).

**Figure 3:**
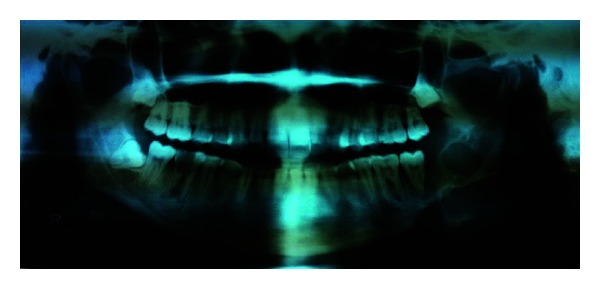
OPG showing multiocular radiolucency in the mandibular left molar-ramus area and discontinuity of the lower border (August 2012).

The patient reported again in August 2012 with a recurrent swelling in the same area. It was associated with difficulty in mastication. There was no history of pain, paresthesia, or discharge. Intraoral examination revealed a proliferative growth measuring 5 × 4 × 3 cms and extending from 35 to retromolar area (Figure 2). Submandibular lymph nodes on the ipsilateral side were enlarged. Orthopantomograph showed multilocular radiolucency involving the left side of the mandible and discontinuity of the lower border (Figure 3). Hemimandibulectomy was performed with primary reconstruction using titanium plate.

**Figure 4:**
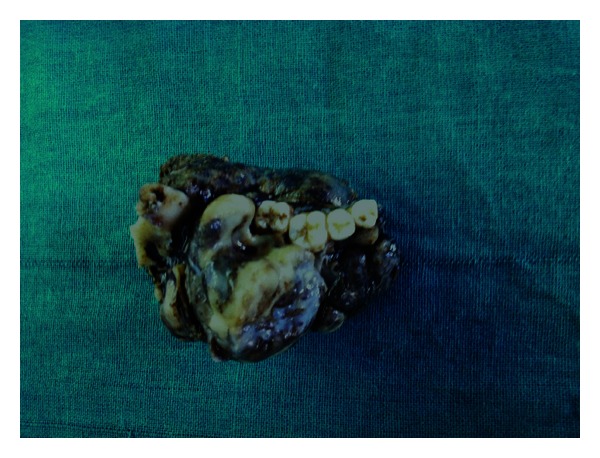
Gross photograph of the left half of the mandible with the tumor mass.

**Figure 5:**
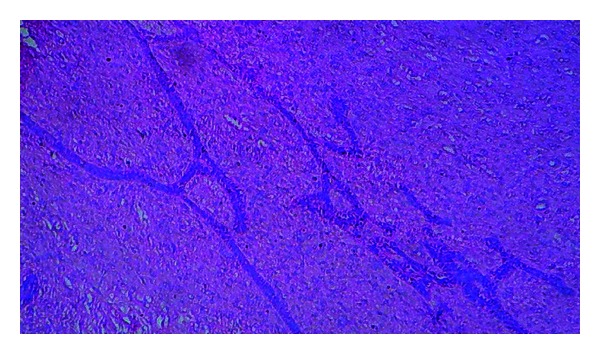
Photomicrograph showing interconnecting strands of odontogenic epithelium in a primitive connective tissue stroma (10x).

**Figure 6:**
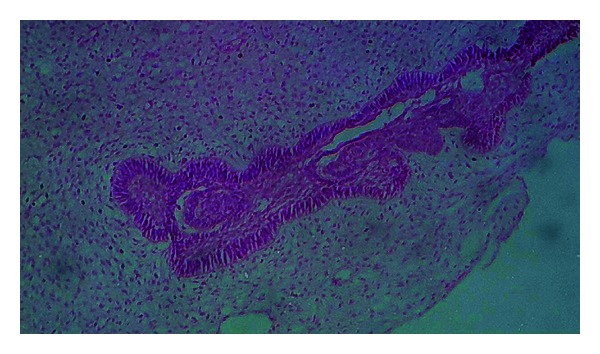
Photomicrograph showing odontogenic epithelial island with peripheral tall columnar cells and central stellate reticulum-like cells in a primitive ectomesenchyme (40x).

**Figure 7:**
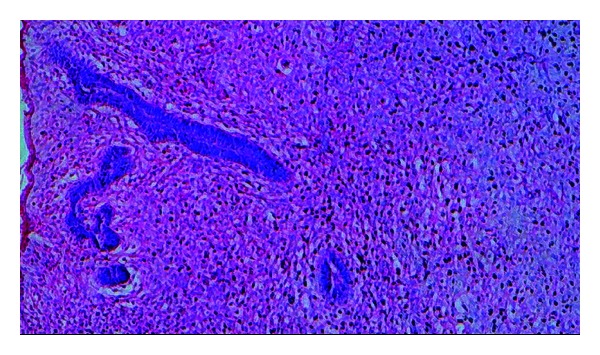
Photomicrograph showing cords and islands of odontogenic epithelium in hypercellular connective tissue stroma (10x).

**Figure 8:**
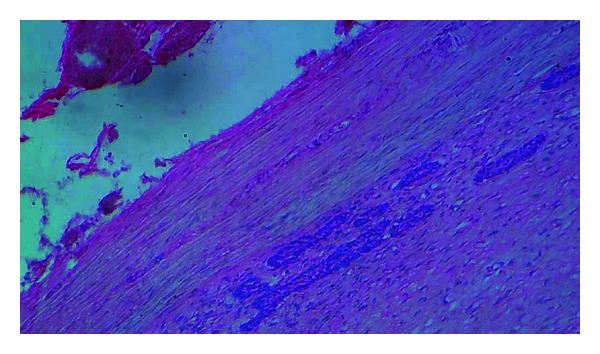
Photomicrograph showing strands of odontogenic epithelium near the capsule (10x).

The excised specimen consisted of left half of the body and ramus of the mandible with the tumor mass measuring 7.5 × 6.5 × 6.5 cms (Figure 4). Histopathologic examination of hematoxylin and eosin-stained sections showed cords, interconnecting strands, and islands of odontogenic epithelium laid in a myxoid cell-rich stroma. The cords and strands consisted of a double layer of cuboidal cells (Figure 5). The islands showed the peripheral tall columnar cells having polarized nucleus, clear vacuolated cytoplasm, and central stellate reticulum-like cells (Figure 6). Juxtaepithelial hyalinization was noted around few islands. There was no hard tissue formation. The degree of cellularity varied in different areas of the lesion. Some areas were hypercellular (Figure 7), whereas others were sparsely cellular and myxoid. Atypia and mitotic activities were not evident. The lesion was partly covered by fibrous capsule (Figure 8). Sections were taken from different areas to look for any malignant changes. However, it was not appreciated. A final diagnosis of ameloblastic fibroma was made.

## 3. Discussion

World Health Organization (WHO 1992) classifies AF as a true mixed neoplasm of odontogenic origin with both epithelial and ectomesenchymal components, without hard tissue formation [2]. It mainly occurs as an intraosseous variant, and only few peripheral cases are reported [9].

Although AF is the most common in posterior mandible region of young adults, a case occurring in anterior mandible of a 45-year-old man with considerable extension and soft tissue involvement has been reported [10].

The exact pathogenesis is not clear. The tall columnar ameloblast-like cells in the epithelial component are too primitive to induce the cells of the ectomesenchyme, and only little is known about their interactions. It is also unknown why induction of odontoblastic differentiation is lacking in AF [2]. Immunohistochemical analysis shows positive staining of odontogenic epithelium for cytokeratin, mesenchymal tissue around the dental lamina-like epithelium for tenascin, focal areas of immature dental papilla-like cells, and basement membrane of the epithelium for vimentin. These findings suggest that AF develops at an early stage of tooth formation [1].

Histological variants of AF that have been described are granular cell AF, in which the ectomesenchyme is dominated by granular cells, papilliferous AF showing marked proliferation of the epithelium with plexiform arrangement [2], ameloblastoma in association with AF, and cystic AF [5]. The density of collagen fibers in AF was found to have an impact on the shape and direction of enlargement of the epithelial follicle. Its growth was found to be restricted in areas of dense collagen deposits, leading to its enlargement in planes of less resistance [11]. In the current case, strands, cords, and islands of odontogenic epithelium are seen in a cell-rich ectomesenchyme. Strands show double layer of cuboidal cells, and islands are lined by tall columnar cells with polarized nucleus surrounding stellate reticulum-like cells. Few islands were bordered by juxtaepithelial hyalinization. Our case presented with an uncommon feature of hypercellularity in few areas which are usually not seen in routine AF. There was no hard tissue formation, thus eliminating ameloblastic fibroodontoma/fibrodentinoma. Hence, it is important that sections are taken from different parts of the lesion especially in recurrent cases of AF to look for any malignant changes. In the present case, atypical features and mitotic activity were not observed.

MIB-1, a monoclonal antibody against proliferation-associated nuclear antigen, in AF ranged from 2.9 to 7.5% and from 1.5 to 13.5% in the epithelial and mesenchymal components, respectively. These indices were higher in recurrent AF and AFS [12]. Knowledge of malignant potential of ectomesenchyme in AF helps to understand its aggressiveness and to determine appropriate management of these benign tumors to prevent malignant transformation to AFS, which can occur years later [13]. 

Approximately, 45% of AFS is reported to arise from a recurrent AF [7]. Hence, sufficient sampling of the pathological specimen is needed to rule out malignant changes if any, which might have begun in focal areas. Cases undergoing malignant transformation show unequivocal changes in the ectomesenchymal component and complete disappearance of odontogenic epithelium [1]. Immunohistochemical markers could be helpful to distinguish AF and AFS. The mesenchymal component of AF is negative to Ki67, PCNA, and p53, in front of the positivity of AFS [14]. But in our case, no histological changes were noted in the stroma, and mitotic activity was not appreciated. 

Whether AF is to be treated by conservative enucleation or by aggressive modality is still a topic of debate. A recurrence rate of 43.5% and 18% was reported by Trodahl and Zallen, respectively. Gundlach was of the opinion that simple enucleation would not be sufficient for AF [2]. Most of them agree for a conservative surgical approach initially, and a more aggressive excision for recurrent lesions, larger tumors, or those involving maxilla [2, 7]. A long-term followup is recommended [4]. Total excision of extraosseous AF with no recurrence is reported [9]. In our case, a conservative surgical approach was followed initially along with the removal of the impacted tooth. But the case reoccurred a year later after the first surgery. Aggressive treatment of hemimandibulectomy was performed for the second time. We recommend a more aggressive treatment in the initial step to prevent its recurrence and transformation to AFS.
